# An Audit of Patient- and System-Level Factors Related to Prolonged Length of Stay on a Female Inpatient Ward

**DOI:** 10.1192/bjo.2026.11847

**Published:** 2026-06-30

**Authors:** Kishanth Srikathirkamanathan, Sukriti Bazaz, Lucy Wright, Saruka Logathasan, Rikke Albert

**Affiliations:** East London NHS Foundation Trust, London, United Kingdom

## Abstract

**Aims:**

Prolonged length of stay (LoS) on acute psychiatric inpatient wards is associated with increased clinical risk, poorer patient experience, reduced patient flow, and higher healthcare costs. National monitoring frameworks focus on average LoS and the proportion of admissions exceeding defined long-stay thresholds. NHS England policy outlines an ambition for average acute adult mental health inpatient stays of approximately 32 days. A UK-focused systematic review identified factors associated with longer psychiatric inpatient stays, including involuntary admission, psychotic diagnosis, older age, and ethnic minority status. Individual studies have also highlighted systemic and social contributors to prolonged LoS, such as out-of-area placements and accommodation-related discharge delays, alongside clinical factors including psychotropic polypharmacy. Local, service-level analyses using routinely collected clinical data can identify patient- and system-level predictors of prolonged LoS, enabling the development of targeted quality improvement initiatives to reduce avoidable extended inpatient admissions.

**Methods:**

All inpatient admissions to a female acute ward over a 12-month period (January–December 2025) were analysed, with readmissions included as separate episodes. A multivariable logistic regression was conducted examining factors associated with prolonged LoS (≥32 days, in line with current NHS mental health policy and performance monitoring frameworks, vs <32 days). Data was extracted from electronic health records: age, ethnicity, local vs. out-of-area placement, primary discharge diagnosis, detention under the Mental Health Act, presence of incidents during admission, discharge destination, safeguarding processes raised, and psychotropic polypharmacy (≥2 concurrent antipsychotics or ≥2 psychotropic medications on discharge)

**Results:**

161 records were included in the analysis. Mean LoS was 29.76 days (SD=29.71). Median LoS was 17 days (IQR 9-31). Forty admissions (24.8%) met criteria for prolonged LoS (≥32 days). Adjusting for all other factors included in the model, prolonged LoS was independently associated with detention under the Mental Health Act (aOR 6.07, 95% CI 2.06–17.88; p=0.001), schizophrenia, schizotypal and delusional disorders (aOR 5.55, 95% CI 1.14–27.00; p=0.034), and mood disorders (aOR 4.77, 95% CI 1.01–22.52; p=0.049). Incidents, safeguarding, polypharmacy, out-of-area placement, discharge destination, ethnicity, and age were not significantly associated with prolonged LoS after adjustment (all p>0.05)

**Conclusion:**

Prolonged inpatient length of stay was independently associated with detention under the Mental Health Act and diagnoses of schizophrenia-spectrum and mood disorders. No other patient- or system-level variables remained significant following multivariable adjustment. Targeted early intervention and proactive care planning for high-risk groups may help reduce avoidable prolonged inpatient stays while maintaining patient safety

